# Short-term evolution of Shiga toxin-producing *Escherichia coli* O157:H7 between two food-borne outbreaks

**DOI:** 10.1099/mgen.0.000084

**Published:** 2016-09-08

**Authors:** Lauren A. Cowley, Timothy J. Dallman, Stephen Fitzgerald, Neil Irvine, Paul J. Rooney, Sean P. McAteer, Martin Day, Neil T. Perry, James L. Bono, Claire Jenkins, David L. Gally

**Affiliations:** ^1^​ Gastrointestinal Bacterial Reference Unit, 61 Colindale Avenue, Public Health England, NW9 5EQ London, UK; ^2^​ Division of Infection and Immunity, The Roslin Institute and Royal (Dick) School of Veterinary Studies, University of Edinburgh, EH25 9RG Roslin, UK; ^3^​ Public Health Agency, 12-22 Linenhall St, BT2 8BS Belfast, Northern Ireland; ^4^​ Microbiology Laboratory, Royal Victoria Hospital, BT12 6BA Belfast, Northern Ireland; ^5^​ U.S. Meat Animal Research Center, Agricultural Research Service, U.S. Department of Agriculture, Clay Center, Nebraska 68933-0166, USA

**Keywords:** Escherichia coli, Bioinformatics, Evolution, Prophage, Recombination

## Abstract

Shiga toxin-producing *Escherichia coli* (STEC) O157:H7 is a public health threat and outbreaks occur worldwide. Here, we investigate genomic differences between related STEC O157:H7 that caused two outbreaks, eight weeks apart, at the same restaurant. Short-read genome sequencing divided the outbreak strains into two sub-clusters separated by only three single-nucleotide polymorphisms in the core genome while traditional typing identified them as separate phage types, PT8 and PT54. Isolates did not cluster with local strains but with those associated with foreign travel to the Middle East/North Africa. Combined long-read sequencing approaches and optical mapping revealed that the two outbreak strains had undergone significant microevolution in the accessory genome with prophage gain, loss and recombination. In addition, the PT54 sub-type had acquired a 240 kbp multi-drug resistance (MDR) IncHI2 plasmid responsible for the phage type switch. A PT54 isolate had a general fitness advantage over a PT8 isolate in rich medium, including an increased capacity to use specific amino acids and dipeptides as a nitrogen source. The second outbreak was considerably larger and there were multiple secondary cases indicative of effective human-to-human transmission. We speculate that MDR plasmid acquisition and prophage changes have adapted the PT54 strain for human infection and transmission. Our study shows the added insights provided by combining whole-genome sequencing approaches for outbreak investigations.

## Data Summary

1. Short read FASTQ sequences have been deposited in the NCBI Short Read Archive under the BioProject PRJNA248042 (http://www.ncbi.nlm.nih.gov/bioproject/248042).
2. Long read FASTA files are deposited in NCBI Genbank under accessions CP015831 (644-PT8 chromosome) (http://www.ncbi.nlm.nih.gov/nuccore/Cp015831), CP015832 (180-PT54 chromosome) (http://www.ncbi.nlm.nih.gov/nuccore/CP015832) and CP015833 (180-PT54 plasmid) (http://www.ncbi.nlm.nih.gov/nuccore/CP015833).

## Impact Statement

In this article, we explore the changes in the genome of a strain of STEC O157:H7 that caused two food-borne outbreaks associated with the same restaurant that were only 8 weeks apart. Utilising sequence data from three different sequencing platforms we provide evidence of short-term evolution between strains isolated in the two outbreaks. This included multi-drug resistance plasmid acquisition and phage content variation, including duplication, that has occurred since the outbreak isolates diverged from a common ancestor over an estimated 1-year period. Based on growth and competitive index assays, we speculate that the genomic changes may account for the higher number of cases associated with the second outbreak. This work has highlighted the value of combining different sequencing and *in vitro* approaches to assist investigations into the epidemiology of outbreaks.

## Introduction

Shiga toxin-producing *E. coli* (STEC) serogroup O157:H7 can cause severe bloody diarrhoea and haemolytic uraemic syndrome and has been a significant public health threat since it emerged in 1982 (Pennington, 2010). Several key virulence factors contribute to pathogenicity; these include the production of Shiga toxin (Stx) and expression of a type three secretion system (Law, 2000). STEC O157:H7 is a globally disseminated pathogen and emerged approximately 120 years ago ( Dallman *et al.*, 2015a).

STEC O157:H7 is a zoonotic pathogen and transmission is commonly associated with direct or indirect contact with animals, especially ruminants, their environment, or the consumption of contaminated food or water. Foodborne outbreaks have been linked to fast-food outlets and restaurants (Bell *et al.*, 1994). Historically, outbreaks were detected and investigated by comparing phage type, pulsed-field gel electrophoresis patterns or multilocus variable number tandem repeat analysis (MLVA) (Byrne *et al.*, 2014). Phage typing has been used for surveillance and outbreak investigations at the Gastrointestinal Bacteria Reference Unit (GBRU) in the United Kingdom since 1992 (Khakhria *et al.*, 1990) and provides a low-cost and rapid test to broadly discriminate between strains. More recently, whole-genome sequencing (WGS) has been used to facilitate STEC O157:H7 outbreak investigations, and strains exhibiting less than five single-nucleotide polymorphisms (SNPs) in the core genome are likely to be temporally linked and share a common source (Dallman *et al.*, 2015b). High-resolution genome-based methods have been use to track human infections phylogenetically (Eppinger *et al.*, 2011; Jenkins *et al.*, 2015).

The sequencing of the Sakai and EDL933 genomes (Hayashi *et al.*, 2001; Latif *et al.*, 2014) showed that both genomes contained an array of integrated prophages with variation of the prophage content between the two strains. Prophages containing the Shiga toxin-encoding genes (*stx*) are known to exhibit variation between STEC O157 strains (Allison, 2007; Eppinger *et al.*, 2011; Herold *et al.*, 2004; Ogura *et al.*, 2015). Little is known however about the short-term micro-evolution of STEC O157:H7 genomes, for example during outbreaks. In part, this is due to difficulties with the assembly of repetitive and paralogous features of prophages when using short-read sequencing caused by multiple assignment in the genomes when reads do not span repeated paralogous prophage genes. Long-read sequencing technologies, such as PacBio or MinION, have been shown to achieve improved *de novo* assemblies that facilitate more accurate characterization of the accessory genome (Cooper *et al.*, 2014; Latif *et al.*, 2014) including prophage regions (Asadulghani *et al.*, 2009).

This study describes a public health investigation of two related outbreaks of STEC O157:H7 associated with the same food outlet in 2012 for which an outbreak report has recently been released (http://www.publichealth.hscni.net/publications/report-outbreak-control-team-investigations-outbreak-e-coli-o157-associated-flicks-rest). In August 2012, four cases of STEC O157:H7 phage type (PT) 8 were epidemiologically-linked to the consumption of food at this restaurant. Eight weeks later, in October 2012, over 140 confirmed cases (and >160 unconfirmed cases) of STEC O157:H7 PT54 were also linked to the same restaurant, with 15 confirmed cases being the result of secondary transmission. MLVA profiles from both incidents indicated that the August and October outbreaks were caused by the same strain despite the phage typing difference. Both short- and long-read sequencing was used to characterize the micro-evolutionary events that occurred in the core and accessory genome between the first (PT8) and the second (PT54) clusters of cases. Our research focused on isolate variation between the two related outbreaks in order to try and understand the much larger scale of the second (PT54) outbreak. The work has facilitated significant insights into short-term changes that can occur in the STEC O157:H7 genome associated with human infection and provides important lessons for outbreak investigations involving this zoonotic pathogen.

## Methods

### PT and MLVA analysis.

All 145 cultures, received at the reference laboratory, from cases linked to both the August and October clusters were typed by phage typing and MLVA (Byrne *et al.*, 2014; Khakhria *et al.*, 1990). The MLVA profiles for the outbreak strains isolated in August and October were mostly 5-8-12-4-5-2-8-3. However, the profile sshowed a high degree of variation at VNTR locus #3 but all isolates were a single-locus variant (SLV) of each other. Isolates that have the same MLVA profile or SLV of that profile are regarded as microbiologically linked (Byrne *et al.*, 2014). MVLA analysis had revealed that isolates from both the PT54 and PT8 outbreaks clustered together and that, despite their different PTs, the two occurrences of STEC O157:H7 at the food outlet were likely to be associated with very closely related strains.

### Illumina sequencing and core SNP analysis.

As part of the outbreak investigation 89 isolates were selected for WGS, including four from the August PT8 cluster and 53 from the PT54 cluster in October and 30 isolates of STEC O157:H7 from temporally and geographically related sporadic cases isolated between June and November 2012. Genomic DNA was fragmented and tagged for multiplexing with Nextera XT DNA Sample Preparation Kits (Illumina) and sequenced at the Animal Laboratories and Plant Health Agency using the Illumina GAII platform with 2×150 bp reads. Short reads were quality trimmed (Bolger *et al.*, 2014) and mapped to the reference STEC O157 strain Sakai (Genbank accession BA000007) using BWA-SW (Li & Durbin, 2009). The sequence alignment map output from BWA was sorted and indexed to produce a binary alignment map (BAM) using Samtools (Li & Durbin, 2009). GATK2 (McKenna *et al.*, 2010) was used to create a variant call format (VCF) file from each of the BAMs, which were further parsed to extract only SNP positions which were of high quality [mapping quality (MQ)>30, depth (DP)>10, genotype quality (GQ)>30, variant ratio >0.9]. Pseudosequences of polymorphic positions were used to reconstruct maximum-likelihood trees using RaxML (Stamatakis, 2014). Pair-wise SNP distances between each pseudosequence were calculated. Spades version 2.5.1 (Bankevich *et al.*, 2012) was run using careful mode with kmer sizes 21, 33, 55 and 77 to produce *de novo* assemblies of the sequenced paired-end fastq files. FASTQ sequences were deposited in the NCBI Short Read Archive under the BioProject PRJNA248042.

### PacBio sequencing.

One isolate of STEC O157 PT8 (ref 644-PT8) and one belonging to PT54 (ref 180-PT54) were selected. High-molecular-weight DNA was extracted using Qiagen Genomic-tip 100/G columns and a modification of the protocol previously described by Clawson *et al.* (2009). Samples (10 µg) of DNA was sheared to a targeted size of 20 kb using a g-TUBE (Corvaris) and concentrated using 0.45×volume of AMPure PB magnetic beads (Pacific Biosciences) following the manufacture’s protocol. Sequencing libraries were created using 5 µg of sheared DNA and the PacBio DNA SMRTbell Template Prep Kit 1.0 and fragments 10 kb or larger selected using a BluePippin (Sage Science) with the smrtbell 15–20 kb setting. The library was bound with polymerase P5 followed by sequencing on a RS II sequencing platform (Pacific Biosciences) with chemistry C3 and the 120 min data collection protocol.

A fastq file was generated from the sequencing reads using SMRTanalysis and error-corrected reads were created using PBcR with self-correction (Koren *et al.*, 2013). The longest 20× coverage of the corrected reads were assembled with Celera Assembler 8.1. The resulting contigs were polished using Quiver (Chin *et al.*, 2013) and annoted using PROKKA (Seemann, 2014). The annotated genome sequence was imported into Geneious (Biomatters) and duplicated sequence removed from the 5′ and 3′ ends to generate the circularized chromosome. The origin of replication was approximated using OriFinder (Luo *et al.*, 2014) and the chromosome reoriented using the origin as base 1. PacBio sequenced strain 180-PT54 is available under accession numbers CP015832 for the chromosome and CP015833 for the plasmid.

### MinION sequencing.

DNA from 644-PT8 was extracted using the STRATEC molecular invisorb spin minikit and diluted to a concentration of 1 µg of genomic DNA in 50 µl of water. The MinION library was prepared using the SQK-MAP006 genomic sequencing kit according to the manufacturer’s instructions and sequencing was performed on a Mk1 MinION with a Mk1 flow cell.

Approximately 76 000 reads were produced and 26-fold passing 2D coverage of the genome was achieved. The long-read assembly program Canu version 1.1 (Koren *et al.*, 2013) was used to assemble the long reads and two chromosomal contigs were produced. The assembly of 644-PT8 showed greater concordance with the synteny of 180-PT54 than the assembly of the PacBio sequencing had produced, but was still not resolved in a similar region.

OpGen mapping for the isolate was obtained from a commercial provider. 644-PT8 was rotated using OriFinder to have the same point of origin as isolate 180-PT54 for comparison. The genome was then annotated using PROKKA (Seemann, 2014). MinION sequenced strain 644-PT8 is available under the accession number CP015831.

### Analysis of the accessory genome using long-read sequences.

The two annotated chromosome assemblies were analysed in PHAST (Zhou *et al.*, 2011) which identifies complete and incomplete prophage regions and their constituent genes.

The gene annotations and their sequences for each strain were compared with each other with blastn (Altschul *et al.*, 1990). Unique genes were extracted from the results if they had no match at greater than 70 % nucleotide identity and overlap to any of the annotated genes in the other strain using a reciprocal blast approach. Roary (Page *et al.*, 2015) was used to confirm whether the identified genes were representative of the rest of the outbreak, i.e. that PT54-specific genes were missing from all PT8 isolates and PT8-specific genes were missing from all PT54 isolates.

NUCMER (Kurtz *et al.*, 2004) was used to align the two assemblies and to identify SNPs, 23 012 ambiguous alignment SNPs were excluded. These SNPs were confirmed by aligning the Illumina-sequenced contigs also to confirm that the SNPs were found with both short -and long-read techonologies.

Gene annotations were extracted from the genbank file and resistance annotations were manually analysed for differences between the two strains. Plasmid differences were visualised in Mauve (Darling *et al.*, 2010). The plasmid from isolate 180-PT54 was graphically visualised using BRIG (Alikhan *et al.*, 2011) and compared with other IncHI2 plasmid sequences found in Genbank that were highly similar by blast at >98 % identity and >60 % coverage (accession numbers KM877269.1, JN983042.1, BX664015.1, DQ517526.1, EF382672.1, LN794248.1, LK056646.1, EU855787.1, KP975077.1, EU855788.1, CP011601.1, CP008906.1, CP008825.1, CP012170.1 and CP006056.1).

### Plasmid conjugation.

Nalidixic-acid-resistant (NalR) colonies of 644-PT8 were isolated from overnight cultures on LB-agar with 20 µg nalidixic acid ml^−1^. Conjugation was performed on LB-agar by co-streaking donor 180-PT54 and recipient spontaneous NalR of 644-PT8. Co-streaked growth was harvested in phosphate-buffered saline then plated onto LB-agar with 20 µg nalidixic acid ml^−1^ and 10 µg chloramphenicol ml^−1^. Resistant colonies were purified by streaking onto fresh plates of 20 µg nalidixic acid ml^−1^ and 10 µg chloramphenicol ml^−1^.

### Acid-resistance assays.

Acid-resistance assays were performed as described previously (Castanie-Cornet *et al.*, 1999). Briefly, cells were cultured overnight in either LBG [Luria-Bertani (LB) broth + 0.4 % glucose], LB buffered with 100 mM morpholinepropanesulfonic acid (MOPS pH8) or LB buffered with 100 mM morpholineethanesulfonic acid (MES pH 5.5). Overnight (22 h) stationary-phase cultures were diluted 1:1000 into pre-warmed minimal E glucose (EG) media, pH 2.5. The glutamate- and arginine-dependent systems were tested by growing cells overnight in LBG and diluting cultures into EG (pH 2.5) supplemented with either 1.5 mM glutamate or 0.6 mM arginine, respectively. The glucose-repressed system was tested by growing cells overnight in LB-MES pH 5.5 followed by dilution in EG pH 2.5. Overnight cultures grown in either LB-MOPS (pH 8) or LBG followed by dilution in unsupplemented EG were used as acid-sensitive controls for the glucose-repressed and glutamate- or arginine-dependent AR systems, respectively. Viable cells were enumerated at t=0 and t=4 h and used to calculate percentage survival.

### Fitness assays.

Fitness of 180-PT54 relative to 644-PT8 was calculated as described previously (Lenski, 1991). Viable-cell counts for each competing strain were determined at time zero (t=0) and again after 24 h of co-culturing by selective plating. Fitness was calculated using the formula:

Fitness index (f.i.) = LN (N_i_ (1)/ N_i_ (0)) **/** LN (N_j_ (1)/ N_j_ (0)),

Where N_i_ (0) and N_i_ (1) = initial and final colony counts of strain 180-PT54, respectively and

N_j_ (0) and N_j_ (1) = initial and final colony counts of strain 644-PT8, respectively (Lenski, 1991).

### Biolog phenotyping microarray.

A single isolated Shiga toxin-containing *Escherichia coli* O157:H7 colony was grown on BUG+B agar overnight at 33 °C. A sterile swab was used to transfer cells from the plate into inoculating fluid 0 (IF-0) to a turbidity of 43 % T (transmittance) and addition IF-0 with dye was to a final cell density of 85 % T. For phenotyping microarray (PM) plates 1 and 2 (Biolog), 100 µl per well was added. PM plates 3, 4, 6, 7 and 8 were supplemented with 20 mM sodium succinate and 2 µM ferric citrate before 100 µl was added to each well (Bochner *et al.*, 2001). All plates were incubated at 33 °C for 48 h using the Omnilog II Combo System (Biolog). The output data from the Omnilog was imported into the opm package in R for analysis (Vaas *et al.*, 2013).

## Results

### Short-read sequencing analysis demonstrates that the outbreak strains are closely related and were not endemic

**Fig. 1.**
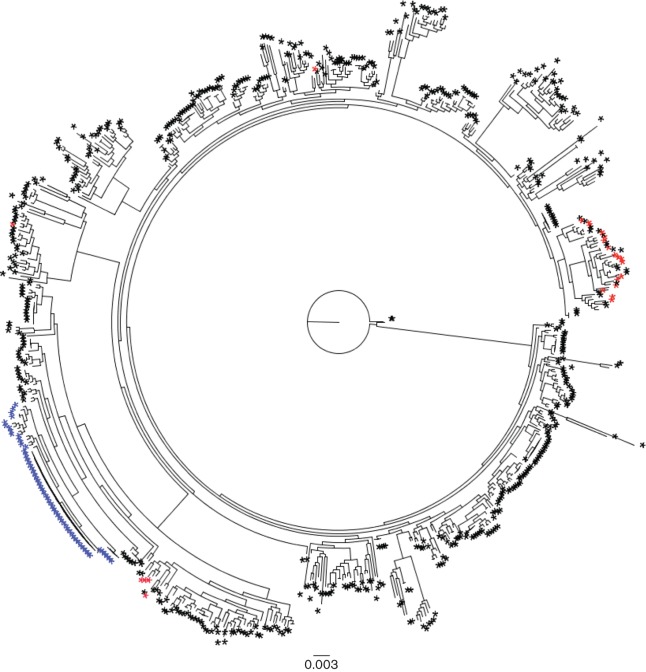
Maximum-likelihood phylogenetic tree of STEC O157 strains selected for various PTs in PHE database associated with domestically acquired infection and travel-related cases. SNPs called on core genome via a mapping technique against the reference strain Sakai. Outbreak strains are indicated in blue and local background strains in the region where the outbreak occurred are in red.

**Fig. 2.**
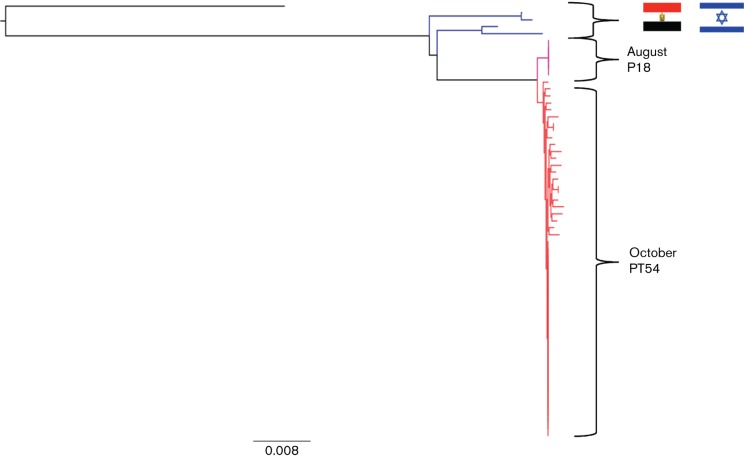
Maximum-likelihood phylogenetic tree of outbreak strains and most closely related strains in the PHE database that are associated with foreign travel to Egypt and Israel. The blue branches represent strains that are associated with travel to Egypt and Israel, the pink branches represent the original PT8 outbreak and the red branches represent the later PT54 outbreak.

Analysis of Illumina sequences indicated that all four isolates from the August PT8 outbreak had identical core genome sequences. There were three SNP differences in the core genome between the PT8 isolates from August and the PT54 isolates from October. The maximum distance between isolates within the October PT54 cluster was four SNPs, including acquisition of a maximum of two SNPs from a common haplotype. Previous temporal analysis of STEC WGS data predicts a mutation rate of approximately 2.5 SNPs per year, therefore it was likely their last common ancestor was very recent (approximately 1 year) but prior to the occurrence of the two public health incidents (Dallman *et al.*, 2015a). The phylogeny of the outbreak isolates indicated that the PT54 cluster did not directly evolve from the PT8 cluster but instead that they share a very recent common ancestor. Furthermore, it was evident that the PT8 and PT54 strains were closely related to each other but genetically distinct from strains of STEC O157:H7 circulating in the local population (Figs 1 and 2). Strains held in the Public Health England (PHE) STEC O157 WGS database that clustered most closely with the outbreak strains were associated with foreign travel to Egypt and Israel (Fig. 2). Although the precise source was never identified by the investigation that followed it was likely that the strains were imported in contaminated food with the larger outbreak possibly exacerbated by an infected or colonised food handler in the restaurant.

### Prophage variation identified between specific PT8 and PT54 isolates based on combined long-read sequencing approaches

PacBio sequencing enabled assemblies of the genome of 180-PT54 into one contig and the genome of 644-PT8 into two contigs. Difficulties with the assembly of 644-PT8 were a result of the reorganized synteny of the genome compared with the closely related PT54 isolate. From comparison of the MinION assembly and the OpGen map, it was clear that the disrupted assembly was caused by a 200 kbp inverted repeat in the genome that constituted the second smaller contig in the assembly. The contig was inserted twice into the larger contig aligning with the NCoI sites found in the OpGen map (Fig. S1, available in the online Supplementary Material). The combination of the MinION sequencing assembly and the OpGen map enabled us to construct a single contig of 5.8 Mb for the isolate that included the 200 kbp repeat.

**Fig. 3.**
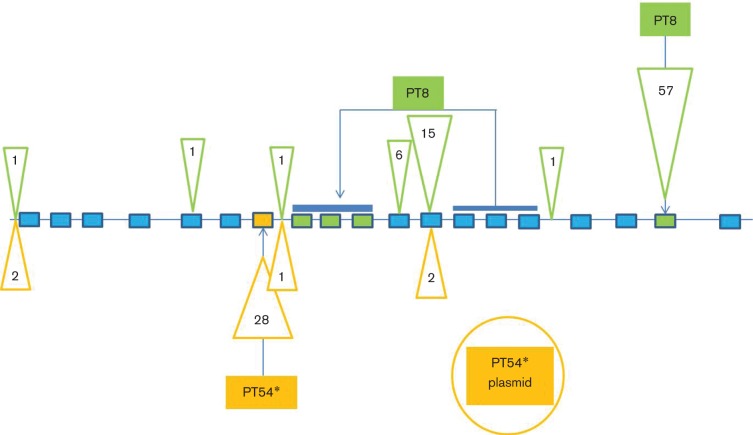
Schematic diagram representing accessory genome variation between the two long-read-sequenced outbreak isolates (180-PT54 and 644-PT8). Blue rectangles represent shared prophage regions, the orange rectangle represents a unique 180-PT54 prophage region and green rectangles represent unique 644-PT8 prophage regions. Orange triangles represent locations and number of unique genes for 180-PT54. The inverted repeat region is indicated by a blue line above the repeated prophage blocks. The unique plasmid in the PT54 outbreak is represented by the orange circle. Those changes that have been confirmed to be representative of all the other members of that PT sub-cluster by Roary analysis have an asterisk next to them.

A set of 14 prophage regions was shared between the representative isolates of the PT8 and PT54 clusters. In addition to the 14 shared prophage regions, 180-PT54 had gained one prophage region of 24 874 bp located at 2 281 433–2 306 307 bp and 644-PT8 had acquired one prophage region of 20 818 bp located at 4 773 172–4 793 990 bp. However, the subsequent Roary analysis showed that the PT8 unique prophage was not missing from all the PT54 outbreak isolates so was not specific to the PT8 outbreak. The 180-PT54 unique prophage was likely to be specific to the PT54 outbreak as it was missing from all the PT8 isolates. In addition, the genome of 644-PT8 had three repeated prophages within the 200 kbp inverted repeat. Two shared prophage regions, designated P7 and P8 were similar prophages that showed variation between the two representative isolates, indicative of recombination that had contributed to the inverted repeat (Fig. S2). The prophage changes between long-read sequenced isolate 644-PT8 and 180-PT54 are detailed in Fig. 3 and those changes that are representative of the rest of that PT sub-cluster are indicated by asterisks.

All the unique gene differences within the chromosome of each representative strain are listed in Table S1. Those that were confirmed by Roary to be representative of the rest of that PT outbreak are highlighted in bold type. The genes that vary between 180-PT54 and 644-PT8 are detailed in Fig. 3. 644-PT8 had 82 unique genes on the chromosome, 79 of which were prophage-associated but the Roary analysis showed that these changes were only present in a subset of the PT54 isolates and not all members of the rest of the PT54 outbreak. 180-PT54 had 30 unique genes, 25 of which were prophage-associated and these were found to be missing from all the other PT8 isolates in the outbreak and therefore were representative of the PT54 outbreak.

**Table 1.** Table listing the positions and base changes of all the SNPs found between the PT8 strain and the PT54 strain in a whole-genome alignment using the program NUCMER The SNPs identified by the previous phylogenetic analysis using Illumina data are highlighted in bold and italicized type, the third SNP identified by the phylogenetic analysis is within a repeat region so was excluded as ambiguous alignment by the program NUCMER. All other identified SNPSs, not in bold, are part of the mobilome.

PT54 position	PT54 base	PT8 base	PT8 position
1975308	G	C	1974495
2681706	C	A	3282472
2681715	T	C	3282463
2681722	T	C	3282456
2681730	T	G	3282448
2681757	T	G	3282421
2681766	G	T	3282412
2681775	A	G	3282403
2681784	C	T	3282394
2681788	G	A	3282390
2681796	T	C	3282382
2681823	G	A	3282355
2681833	G	A	3282345
2681835	C	T	3282343
2681844	A	G	3282334
2681847	G	A	3282331
2681865	G	A	3282313
2681973	C	T	3282205
2681976	C	G	3282202
2681977	T	C	3282201
2681979	T	A	3282199
2681981	A	C	3282197
2681982	C	A	3282196
2681985	C	A	3282193
***2833323***	***A***	***C***	***3027880***
***2894348***	***A***	***G***	***3088905***
3048043	C	A	3242600
3048059	C	A	3242616
3048069	G	A	3242626

Whole-genome alignment using NUCMER identified 29 SNPs between the two isolates (644-PT8 and 180-PT54) in fully aligned regions. The SNP locations and base changes are detailed in Table 1 and have been confirmed in the Illumina data. This was higher than the three SNPs identified between the ‘core’ genomes based on the short-read sequencing but 26 of these SNPs were found in the P7 and P8 shared prophage regions. These prophage regions would not have been shared with Sakai so would not have been called in the original core genome SNP-calling from the Illumina data.

### Plasmid acquisition by 180-PT54

**Fig. 4.**
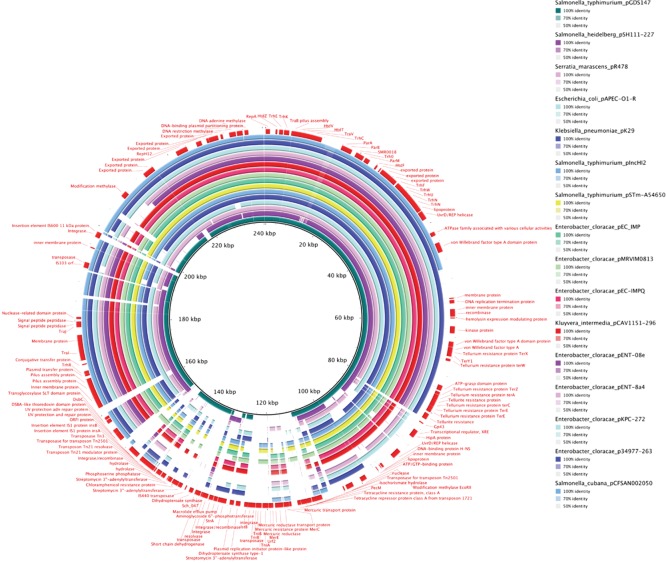
BRIG plot of the approximately 240 kbp IncHI2 plasmid found in 180-PT54 as the central reference showing genomic similarity between it and other IncHI2 plasmids found in Genbank from various species of bacteria. Annotations are shown in red on the outermost ring. The darker the colour the greater the level of genomic similarity between the 180-PT54 IncHI2 plasmid and the plasmid found in a different organism. The plasmids are indicated by the colours labelled vertically at the right hand side of the figure.

Both strains harboured pO157, the O157 virulence plasmid present in nearly all strains of STEC O157:H7 (Lim *et al.*, 2010). However, isolate 180-PT54 acquired an additional approximately 220 genes introduced on an IncHI2 plasmid (Johnson *et al.*, 2006) not present in 644-PT8. While both 180-PT54 and 644-PT8 exhibited tellurite and tetracycline resistance, 180-PT54 was also resistant to chloramphenicol and streptomycin and this matched with resistance genes located on the IncHI2 plasmid. The IncHI2 plasmid was predicted to encode at least six membrane proteins, a drug efflux pump, other resistance mechanisms including additional tellurite resistance and protection from exposure to heavy metal ions (mercury). It also encoded at least two DNA methylases (Fig. 4). Relatedness depicted in a BRIG plot (Fig. 4) demonstrates the high similarity with other IncHI2 plasmids that have been detected in clinical isolates worldwide (Chen *et al.*, 2007; Feasey *et al.*, 2014; Gilmour *et al.*, 2004; Kariuki *et al.*, 2015; Li *et al.*, 2013).

### Phage type transition is associated with plasmid acquisition

It was suggested that the IncHI2 plasmid may be responsible for the difference in PT observed between the two outbreak clusters in August and October. To test this, the IncHI2 plasmid was conjugated into 644-PT8 and the conjugant was then phage typed. Acquisition of the plasmid, as defined by inheritance of chloramphenicol resistance, converted 644-PT8 to PT54. Antibiotic resistance and replicon type profiling using the Illumina Roary data demonstrated that the plasmid-associated resistance was present in all the PT54 isolates in this study and that they all carried the IncHI2 plasmid associated with the PT transition. Analysis of the genes present on the plasmid (Fig. 4) shows a number of determinants that could be associated with changes in phage resistance including tellurite resistance (Whelan *et al.*, 1997). While both 644-PT8 and 180-PT54 have chromosomally-encoded tellurite resistance, specifically *terW*, only 180-PT54 have *terY* and *terX* as these are present on the IncHI2 plasmid. Methylase-modification genes encoded on the plasmid are also potential candidates to confer resistance to specific bacteriophages (Labrie *et al.*, 2010).

### Increased fitness of the PT54 strain associated with the larger second outbreak

Acquisition of IncHI2 plasmids commonly confers phage, antibiotic and heavy metal resistance, thus increasing bacterial fitness under certain environmental conditions (Fang *et al.*, 2016; Whelan *et al.*, 1995). Conversely an increased metabolic burden imposed by the 200 kbp inversion during DNA replication in strain 644-PT8 is likely to reduce fitness. To assess any differences in fitness, strains 644-PT8 and 180-PT54 were competed by co-culturing in LB-broth at 37 °C and 25 °C. Under both conditions 180-PT54 significantly outcompeted 644-PT8 (fitness index=1.28 and 1.23, respectively). Fitness was therefore independent of culture temperature. Subsequently Biolog phenotypic microarrays were performed to determine the nature of the observed fitness increase. Similar growth was observed for both strains for the majority of carbon, phosphorus and sulphur sources tested (data not shown) however growth of 180-PT54 was increased for multiple nitrogen sources, including certain amino acids and dipeptides (Fig. S3). This is in agreement with the observed increased fitness of 180-PT54 when cultured in LB-broth in which amino acids/short peptides are the primary carbon and nitrogen sources. In addition, growth of 180-PT54 was better than that of 644-PT8 when ammonia was the sole nitrogen source (Fig. S3). Of the multiple di- and tri-peptide nitrogen sources on which 180-PT54 grew better than 644-PT8 many contained arginine or glutamate. *E. coli* possesses three acid-resistance systems (AR) of which two are dependent on arginine and glutamate, respectively (Richard & Foster, 2003). We therefore tested if AR was altered in 180-PT54 by the increased metabolism of arginine and glutamate relative to 644-PT8. For each AR system (glucose-repressed, arginine- and glutamate-dependent) strain 644-PT8 was significantly more resistant to acid shock when either pre-adapted in LB pH 5.5 or supplied with exogenous Arg or Glu (Table 2). Without pre-adaptation however strain 180-PT54 was more acid-resistant than 644-PT8 (*P*=0.035).

**Table 2.** Table describing the results of the acid-resistance assays performed on strains 644-PT8 and 180-PT54 to assess their biological fitness

Adaptation medium*	Challenge medium (pH2.5)	Percentage survival^†^	
		PT54	PT8
LB pH8	EG	0.811582004	0.187176
LB pH 5.5	EG	4.09726344	38.78565
LBG	EG	14.77276286	7.230324
	EG+Glu	21.71965529	52.69993
	EG+Arg	37.2807674	70.11365

*Strains were adapted by overnight culturing in either LB pH 5.5 or LBG before diluting 1:1000 in EG pH 2.5 or supplemented EG. ^†^The percentage survival was determined after 4 h of acid challenge. The mean percentage survival of six replicates (*n*=6) is shown for EG challenge and three replicates (*n*=3) for EG supplemented with either glutamate (1.5 mM) or arginine (0.6 mM).

## Discussion

Phylogeny techniques based on ‘core genome’ sequence analyses have been transformative for epidemiological investigations and also provide an assessment of evolutionary relationships between strains (Holt *et al.*, 2012; Quick *et al.*, 2015). This study focused on two temporally related outbreaks of STEC O157:H7 from the same restaurant. Initially, MLVA and phage typing results were contradictory as MLVA indicated that the outbreaks were caused by the same strain although the phage types were distinct. The relatedness of the PT8 and PT54 strains was confirmed by short-read sequencing which defined three SNP differences in the core genome between the two groups of strains indicating that they share a very recent common ancestor (approximately 1 year). Switching of PT within a sublineage has been observed previously (Dallman *et al.*, 2015b) but this is some of the first, to our knowledge, documented evidence of PT conversion within two closely related outbreaks and the mechanisms behind that.

In this study, the application of PacBio and MinION sequencing, as well as OpGen mapping, enabled us to obtain single-contig assemblies of two isolates associated with the two outbreaks at the restaurant, one belonging to PT8 and one belonging to PT54. These assemblies clearly showed the high prophage carriage in these isolates, which is typical of STEC O157:H7 with approximately 12–14 % of the genome made up of highly paralogous phage genes (Fig. S2). Analysis of the genomes from the long-read sequencing showed that there had been a shift in prophage composition between the two outbreak groups. There was gain and loss of prophage while two of the shared prophage regions had undergone recent recombination. Furthermore, isolate 644-PT8 had a repeat of three of the shared prophage regions in a 200 kbp inverted region. There is significant, apparent functional redundancy across the different prophages. This is in agreement with earlier research from Hayashi and colleagues that showed a diverse bacteriophage complement produced from a single strain including recombination between prophage loci (Asadulghani *et al.*, 2009).

Isolate 180-PT54 assembled into three contigs; the chromosome, the F-like pO157 and an IncHI2 plasmid. The IncHI2 plasmid was large (240 kbp) and predicted to encode about 220 genes. This plasmid was not present in 644-PT8. IncHI2 plasmids are commonly associated with the spread of extended-spectrum β-lactam resistance (ESBL) genes, heavy metal resistance and phage resistance (Whelan *et al.*, 1995; Fang *et al.*, 2016). We identified several antibiotic and environmental resistance genes potentially conferring resistance to chloramphenicol, streptomycin, tellurite, tetracycline and certain heavy metal ions encoded on the incHI2 plasmid acquired by 180-PT54 (Fig. 4). The resistance loci facilitated conjugation of the plasmid into 644-PT8, leading to the recipient strain phage typing as PT54. Conversion of PT8 to PT54 is caused by the acquisition of resistance to the group 3 typing phages (TP4, TP5 and TP14) (Cowley *et al.*, 2015). There is a previous report that an IncHI2 plasmid can confer bacteriophage resistance (Whelan *et al.*, 1997) therefore adding to the possible survival advantage conferred on strains that acquire this plasmid. A blast search revealed that highly similar IncHI2 plasmids have been described in several different organisms from around the world (Fig. 4), including Taiwan, China, Kenya, Malawi and the USA. Identification of incHI2 plasmids in *E. coli* however is rare (Fang *et al.*, 2016; Losada *et al.*, 2016). The acquisition of this plasmid is therefore likely to increase the survival capacity of the strain under certain stressful environmental conditions.

In addition to antibiotic and phage resistance, we demonstrated that 180-PT54 was significantly fitter than 644-PT8 under a defined set of growth conditions. The genetic differences identified in 180-PT54, that include plasmid acquisition, resulted in a fundamental alteration in central nitrogen metabolism that enhanced growth compared with 644-PT8. In accordance with the trade-off between self-preservation and nutritional competency (SPANC) the increased growth of 180-PT54 also resulted in decreased acid resistance, at least under priming conditions (Ferenci, 2005). The public health investigation of the outbreaks included sampling of employees who worked at the restaurant. Two members of staff were shown to be colonized with the PT54 strain during the outbreak and an analysis identified a significant association with one of these employees and the infection risk (http://www.publichealth.hscni.net/publications/report-outbreak-control-team-investigations-outbreak-e-coli-o157-associated-flicks-rest), although it is not known if this individual could have actually accounted for the second outbreak. Based on this and the altered genotype and phenotype of the PT54 strain, we speculate that it may be more adapted for human colonization than the original PT8 strain and that this capacity may be linked to the much higher number of cases associated with the second outbreak. These included multiple examples of human-to-human transmission. STEC O157:H7 strains are usually associated with ruminant hosts and presumably human colonization could lead to adaptive changes that promote survival, such as plasmid acquisition and prophage variation. Sequencing of human cases in the UK has identified a subset of imported strains that are acquired during travel abroad or brought in by colonised foreign visitors (Dallman *et al.*, 2015a). These strains are significantly more likely to contain plasmids encoding antibiotic resistance and there is a concern that acquisition of such elements may adapt strains to enable transmission and/or persistence in the human population, which would be a serious public health concern. However, we do acknowledge that there are other possible reasons why a greater number of cases were associated with the second outbreak; these include the possibility of different contaminated products in the second outbreak or increased awareness that might introduce a bias in recorded cases.

This outbreak investigation illustrated the power of both short- and long-read sequencing technologies to investigate and understand foodborne outbreaks. The evolutionary context illustrated by the short-read WGS data revealed the true genetic relationship between the strains from the August and October clusters and provided evidence of the geographical origin of the strains. The geographical signal derived from WGS data will greatly facilitate outbreak investigation where imported food is implicated. The use of long-read WGS data clearly demonstrated the dynamic nature of the accessory genome in STEC O157:H7 and the potential impact of horizontal gene transfer over a short time frame. The long-read sequencing enabled us to identify a plasmid that confers resistance to antibiotics and bacteriophages as well as other environmental stressors. The plasmid was shown to causes phage-type conversion in a strain of STEC O157:H7.

## Supplementary Data

979 Supplementary File 1
