# Supplementary material for: Short-term evolution of Shiga toxin-producing Escherichia coli O157:H7 between two food-borne outbreaks
Source: Microb Genom. 2016 Sep 8;2(9):e000084. doi: 10.1099/mgen.0.000084 (PMC5320650; doi:10.1099/mgen.0.000084)
Supplement: Supplementary File 1 [file mgen-02-84-s001.docx]

**Supplementary materials**

**Table 1.** Table detailing the unique chromosomal genes found in the PT8 and PT54 genes with their annotation and chromosome location. Those genes that distinguish the two outbreaks (found in all isolates of that PT but not the other) are highlighted in bold and italicised.

| **PT8 Unique genes** | **PT8 Genome location** | **PT54 Unique genes** | **PT54 Genome location** |
| --- | --- | --- | --- |
| 644_00713_hypothetical_protein | 722431-722838 | 180_00068_ilvB_operon_leader_peptide | 75339-75428 |
| 644_02073_hypothetical_protein | 2102929-2103033 | 180_00338_hypothetical_protein | 356642-356917 |
| 644_02384_hypothetical_protein | 2375234-2375443 | ***180_02292_Phage_tail_sheath_protein*** | ***2286868-2288052*** |
| 644_02879_hypothetical_protein | 2841585-2841818 | ***180_02293_Phage_tail_tube_protein_FII*** | ***2288052-2288564*** |
| 644_02880_hypothetical_protein | 2841796-2842203 | ***180_02294_Phage_tail_protein_E*** | ***2288619-2288984*** |
| 644_02883_transcriptional_repressor_DicA | 2843080-2843487 | ***180_02295_Phage-related_minor_tail_protein*** | ***2289135-2291225*** |
| 644_02889_hypothetical_protein | 2847420-2848178 | ***180_02296_hypothetical_protein*** | ***2291237-2291938*** |
| 644_02892_hypothetical_protein | 2849217-2849495 | ***180_02297_Phage_P2_GpU*** | ***2291951-2292439*** |
| 644_02895_hypothetical_protein | 2850924-2851454 | ***180_02298_Serine_acetyltransferase*** | ***2292596-2293168*** |
| 644_03322_hypothetical_protein | 3265153-3265803 | ***180_02302_Plasmid_stability_protein*** | ***2294834-2295148*** |
| 644_03323_IpaB/EvcA_family_protein | 3267310-3267900 | ***180_02303_Plasmid_segregation_protein_ParM*** | ***2295153-2296112*** |
| 644_03324_hypothetical_protein | 3268084-3268731 | ***180_02304_Bacteriophage_replication_gene_A_protein_(GPA)*** | ***2296189-2299011*** |
| 644_03325_hypothetical_protein | 3269486-3269755 | ***180_02305_hypothetical_protein*** | ***2299018-2299383*** |
| 644_03329_Transposase,_Mutator_family | 3272657-3273004 | ***180_02306_hypothetical_protein*** | ***2299456-2299686*** |
| 644_03331_Effector_protein_NleF | 3273034-3273291 | ***180_02307_hypothetical_protein*** | ***2300009-2300308*** |
| 644_03333_Tyrosine_recombinase_XerD | 3275358-3276050 | ***180_02308_hypothetical_protein*** | ***2300305-2300571*** |
| 644_03334_hypothetical_protein | 3276711-3278036 | ***180_02309_hypothetical_protein*** | ***2300568-2300771*** |
| 644_03347_hypothetical_protein | 3291283-3291399 | ***180_02310_hypothetical_protein*** | ***2300795-2301211*** |
| 644_03371_hypothetical_protein | 3308835-3309365 | ***180_02311_hypothetical_protein*** | ***2301304-2301417*** |
| 644_03374_hypothetical_protein | 3310794-3311072 | ***180_02312_hypothetical_protein*** | ***2301414-2301656*** |
| 644_03377_hypothetical_protein | 3312111-3312869 | ***180_02313_hypothetical_protein*** | ***2301668-2301946*** |
| 644_03383_transcriptional_repressor_DicA | 3316802-3317209 | ***180_02314_hypothetical_protein*** | ***2301957-2302307*** |
| 644_03386_hypothetical_protein | 3318086-3318493 | ***180_02315_hypothetical_protein*** | ***2302329-2302532*** |
| 644_03387_hypothetical_protein | 3318471-3318704 | ***180_02316_Helix-turn-helix_domain_protein*** | ***2302831-2303235*** |
| 644_03792_hypothetical_protein | 3639232-3639387 | ***180_02317_flagella_biosynthesis_regulator*** | ***2303251-2303901*** |
| 644_04784_hypothetical_protein | 4717144-4717524 | ***180_02318_hypothetical_protein*** | ***2303931-2304278*** |
| 644_04785_hypothetical_protein | 4717566-4718660 | ***180_02319_Tyrosine_recombinase_XerC*** | ***2304284-2305285*** |
| 644_04786_hypothetical_protein | 4718614-4718826 | ***180_02407_hypothetical_protein*** | ***2390892-2391014*** |
| 644_04787_hypothetical_protein | 4719008-4719424 | ***180_03074_hypothetical_protein*** | ***3048749-3048847*** |
| 644_04788_Lactate_utilization_protein_C | 4719919-4720614 | ***180_03076_hypothetical_protein*** | ***3049616-3050173*** |
| 644_04789_Lactate_utilization_protein_B | 4720607-4722034 |  |  |
| 644_04790_Lactate_utilization_protein_A | 4722045-4722764 |  |  |
| 644_04791_Virulence_regulon_transcriptional_activator_VirF | 4723292-4724146 |  |  |
| 644_04792_Mercuric_reductase | 4724372-4725697 |  |  |
| 644_04793_Inner_membrane_protein_YkgB | 4726054-4726647 |  |  |
| 644_04794_putative_oxidoreductase_YtbE | 4726807-4727676 |  |  |
| 644_04795_Right_origin-binding_protein | 4727925-4728782 |  |  |
| 644_04796_Invasin | 4728903-4733156 |  |  |
| 644_04797_Glyoxal_reductase | 4733722-4734297 |  |  |
| 644_04798_hypothetical_protein | 4734490-4734846 |  |  |
| 644_04799_HTH-type_transcriptional_regulator_DmlR | 4735134-4736060 |  |  |
| 644_04800_Alpha/beta_hydrolase_family_protein | 4736217-4737137 |  |  |
| 644_04801_NADH_oxidase | 4737372-4738514 |  |  |
| 644_04805_50S_ribosomal_protein_L31_type_B | 4741899-4742165 |  |  |
| 644_04806_50S_ribosomal_protein_L36_2 | 4742165-4742305 |  |  |
| 644_04807_hypothetical_protein | 4742375-4742566 |  |  |
| 644_04808_HTH-type_transcriptional_regulator_MatA | 4743391-4743933 |  |  |
| 644_04809_hypothetical_protein | 4744008-4744595 |  |  |
| 644_04810_hypothetical_protein | 4744653-4745321 |  |  |
| 644_04811_hypothetical_protein | 4745347-4747872 |  |  |
| 644_04812_hypothetical_protein | 4747862-4748442 |  |  |
| 644_04813_hypothetical_protein | 4749474-4750184 |  |  |
| 644_04814_Inner_membrane_protein_YagU | 4751074-4751688 |  |  |
| 644_04815_Carbon_monoxide_dehydrogenase_small_chain | 4752106-4752795 |  |  |
| 644_04816_4-hydroxybenzoyl-CoA_reductase_subunit_beta | 4752792-4753748 |  |  |
| 644_04817_Xanthine_dehydrogenase_molybdenum-binding_subunit | 4753745-4755943 |  |  |
| 644_04818_XdhC_and_CoxI_family_protein | 4755953-4756909 |  |  |
| 644_04819_Purine_ribonucleoside_efflux_pump_NepI | 4757088-4758215 |  |  |
| 644_04820_Alpha/beta_hydrolase_family_protein | 4758357-4759415 |  |  |
| 644_04821_HTH-type_transcriptional_regulator_DmlR | 4759661-4760563 |  |  |
| 644_04822_hypothetical_protein | 4761266-4761544 |  |  |
| 644_04823_putative_oxidoreductase | 4761711-4762433 |  |  |
| 644_04824_HTH-type_transcriptional_regulator_DmlR | 4762532-4763431 |  |  |
| 644_04825_hypothetical_protein | 4764107-4765063 |  |  |
| 644_04826_DNA_primase_TraC | 4765196-4767529 |  |  |
| 644_04827_hypothetical_protein | 4767543-4767866 |  |  |
| 644_04828_hypothetical_protein | 4767866-4768087 |  |  |
| 644_04829_Ash_protein_family_protein | 47680084-4768641 |  |  |
| 644_04830_Prophage_CP4-57_regulatory_protein_(AlpA) | 4768638-4768898 |  |  |
| 644_04831_Phage_polarity_suppression_protein_(Psu) | 4769832-4770584 |  |  |
| 644_04832_Phage_polarity_suppression_protein_(Psu) | 4770581-4771132 |  |  |
| 644_04833_DNA-binding_transcriptional_regulator | 4771138-4771410 |  |  |
| 644_04834_hypothetical_protein | 4771820-4772836 |  |  |
| 644_04835_hypothetical_protein | 4772386-4772976 |  |  |
| 644_04836_hypothetical_protein | 4773007-4773639 |  |  |
| 644_04837_hypothetical_protein | 4773632-4774090 |  |  |
| 644_04838_hypothetical_protein | 4774090-4774707 |  |  |
| 644_04839_hypothetical_protein | 4774680-4775096 |  |  |
| 644_04840_Putative_prophage_CPS-53_integrase | 4775100-4776281 |  |  |
| 644_04841_IS2_transposase_TnpB | 4776849-4777202 |  |  |
| 644_04842_HTH-type_transcriptional_regulator_YdeO | 4777244-4777987 |  |  |
| 644_04843_hypothetical_protein | 4778811-4779584 |  |  |

**
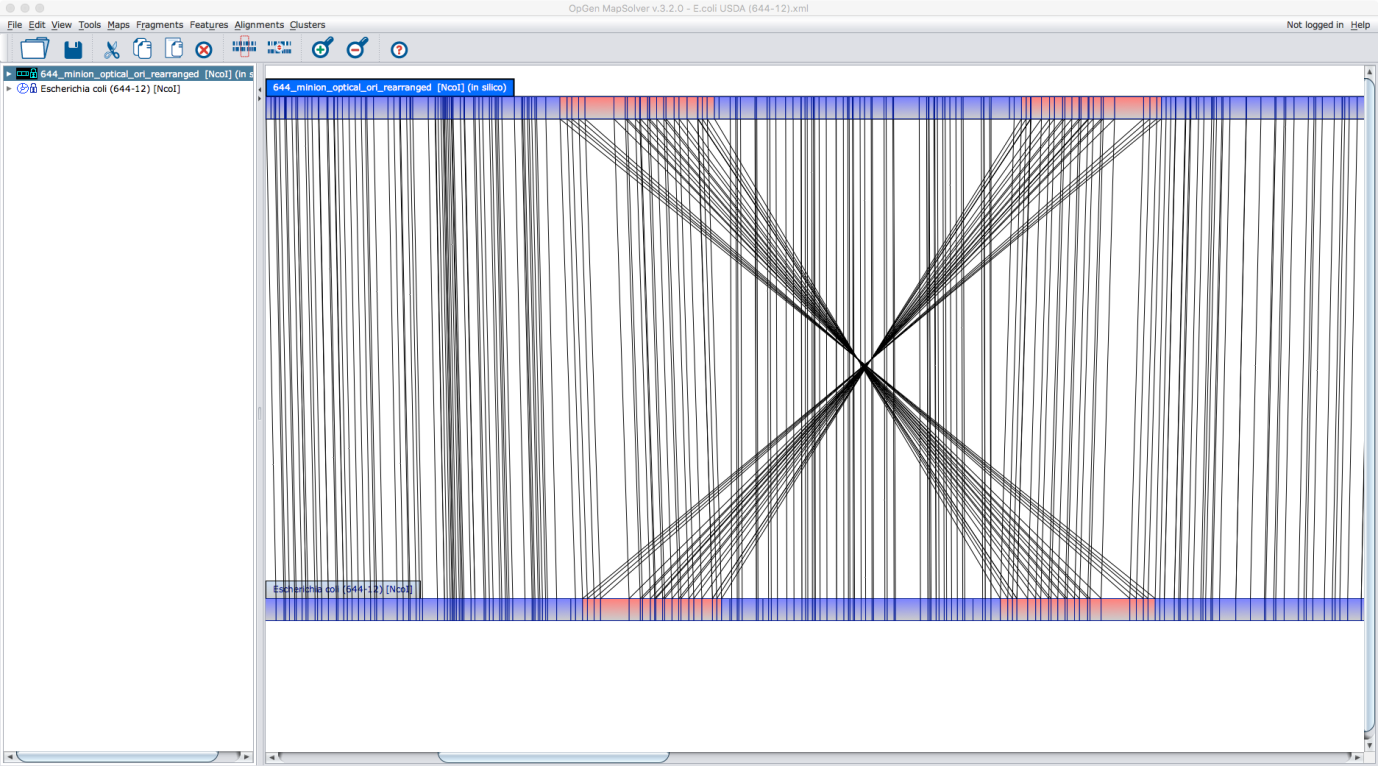
**

**Supplementary Figure 1.** OpGen map alignment with MinION assembly of the PT8 strain with smaller contig inserted twice. The NCol sites are joined up by single black lines clearly showing two regions with the same NCol sites that can each be mapped to two regions. All NCol sites have lined up nicely indicating a correct assembly.


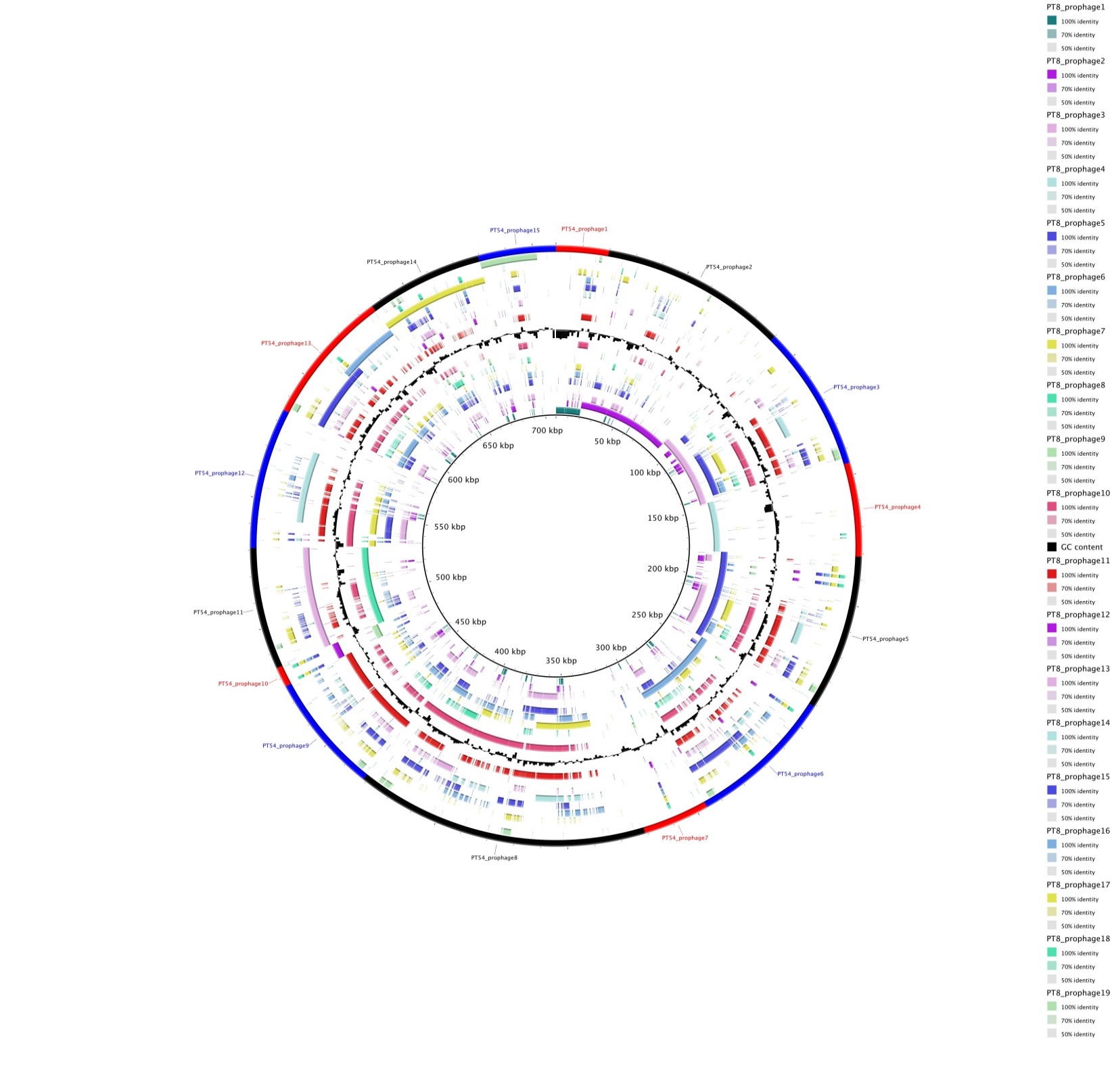


**Supplementary figure 2.**. BRIG plot showing genomic similarity between prophage regions of 180-PT54 and 644-PT8. The central ring shows a Multifasta of 180-PT54 prophage regions (labelled on the outside) and each concentric ring represents 1 prophage region of the PT8 strain (labelled along the right-hand side). The darker the colour the greater the level of genomic similarity in that part of the region. GC content is represented in black in the 11^th^ ring.


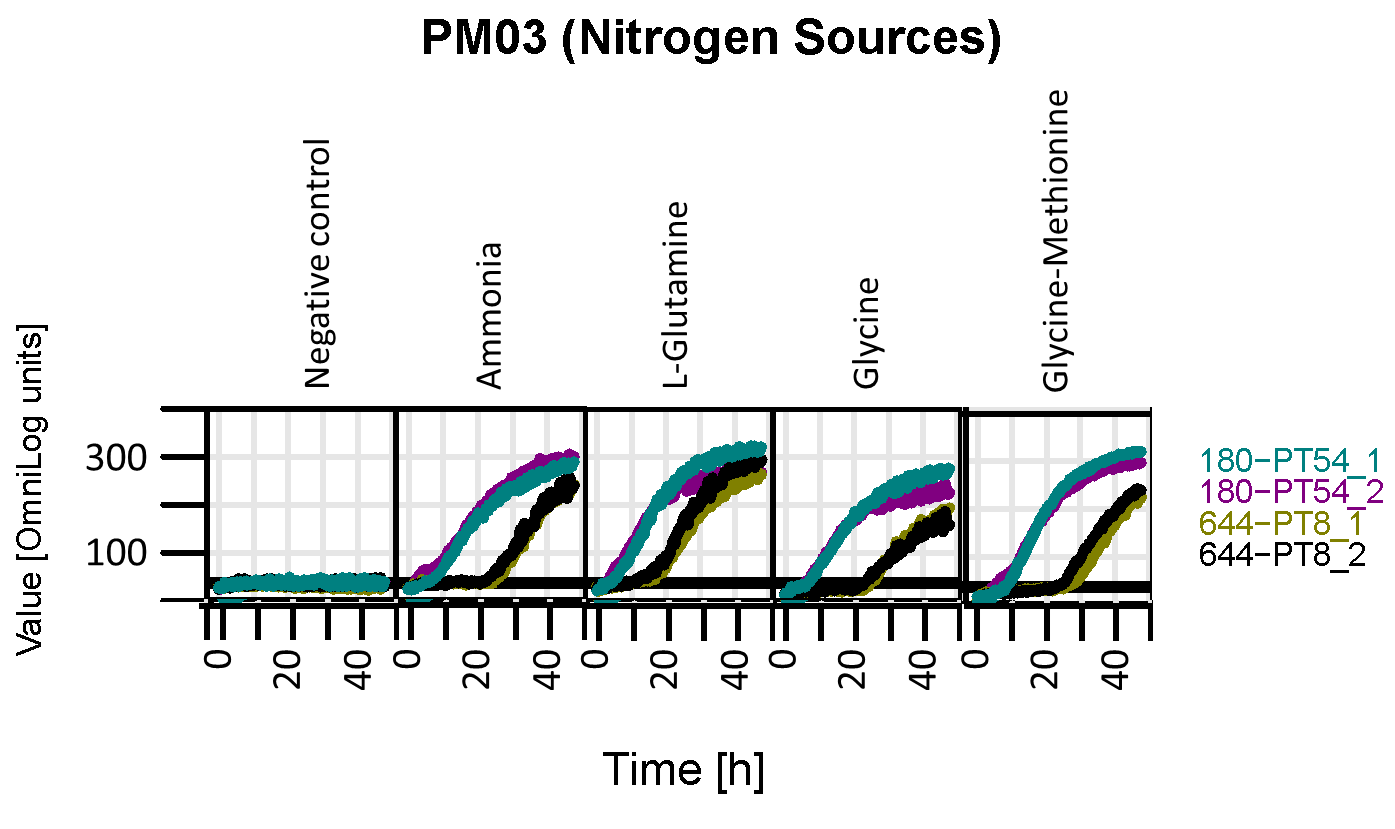


**Supplementary figure 3.** XY Plots of respiration curves from Shiga toxin-containing *Escherichia coli* strains 180 and 644 grown in four nitrogen sources from Phenotype MicroArray plate PM03 and the negative control. Each strain was run in duplicate on different day and indicated by the different colored curves. The growth time in hours is on the x-axis and reduction of the reporter dye was quantified as OmniLog units are represented on the y-axis
